# Dehydration-based preservation of cat cumulus–oocyte complexes is not improved by membrane-permeable trehalose

**DOI:** 10.3389/fvets.2026.1805930

**Published:** 2026-04-07

**Authors:** Pei-Chih Lee, Yuqing Yang, Kaywalee Chatdarong, Pierre Comizzoli

**Affiliations:** 1 Smithsonian’s National Zoo and Conservation Biology Institute, Washington, DC, United States; 2 Department of Obstetrics, Gynaecology and Reproduction, Faculty of Veterinary Science, Chulalongkorn University, Bangkok, Thailand

**Keywords:** dehydration, domestic cat, microwave-assisted drying, oocyte, trehalose hexaacetate, trehalose

## Abstract

**Introduction:**

Dry-preservation of gametes offers a promising alternative to cryobanking by potentially storing cells at ambient temperatures; however, successful preservation of whole oocytes remains limited by dehydration-induced cellular damages. This study evaluated whether intracellular delivery of trehalose via a membrane-permeable derivative, trehalose hexaacetate (6-O-Ac-Tre), could enhance dehydration tolerance of domestic cat cumulus–oocyte complexes (COCs), a relevant model for mammalian fertility preservation.

**Methods:**

Cat COCs were incubated with 0, 3, 10, or 30 mM of 6-O-Ac-Tre to assess intracellular trehalose incorporation and cytotoxicity. Non-cytotoxic doses (3 and 10 mM) were then tested for their ability to mitigate cellular damage following 10 or 15 min of microwave-assisted drying and rehydration, with outcomes including DNA integrity, mitochondrial membrane potential, cell membrane integrity, and meiotic maturation competence.

**Results:**

Overnight exposure to 6-O-Ac-Tre enabled dose-dependent intracellular accumulation of trehalose. However, cytotoxicity examination revealed that prolonged exposure to 30 mM 6-O-Ac-Tre reduced survival and oocytes’ meiotic and developmental competence (*p* < 0.05). Although DNA integrity was largely preserved after drying (*p* > 0.05), meiotic maturation of oocytes was severely compromised (*p* < 0.05). Pre-incubation with 3 or 10 mM 6-O-Ac-Tre did not mitigate (*p* > 0.05) a reduction in mitochondrial membrane potential, cell membrane integrity, and meiotic competence.

**Discussion:**

These findings demonstrated that, while 6-O-Ac-Tre effectively delivered trehalose into COCs, this approach did not improve dehydration tolerance of whole oocytes under the conditions tested. Collective results reflected limitations of the 6-O-Ac-Tre delivery strategy owing to its potential cytotoxicity at higher concentrations. Alternative trehalose delivery conditions or approaches need to be explored to facilitate the development of effective dry-preservation strategies for oocytes.

## 1 Introduction

For women facing medical treatments that may compromise their fertility or those wishing to delay childbearing, fertility preservation offers an opportunity to maintain their reproductive potential. Fertility preservation also benefits agriculture and conservation efforts by safeguarding the genetic diversity of valuable livestock and endangered species. Current fertility preservation strategies involve cryopreserving gametes and gonadal tissues and storing them in liquid nitrogen. Specialized equipment and facilities, as well as continuous supply of liquid nitrogen, are usually required to secure the long-term storage of the biospecimens (1). These requirements may limit the availability of biobanks in regions with more constraints in infrastructural and financial resources.

In the recent decade, long-term storage of gametes in a dried state at ambient temperatures has been explored as a new frontier of fertility preservation (2). When left unprotected, dehydration stress could induce a wide array of injuries within a cell, including membrane fragmentation, decreased mitochondrial health, and DNA damage. Inspired by natural anhydrobiosis of various organisms, dry-preservation techniques utilize disaccharides, such as trehalose, as protective agents to mitigate these detrimental effects (3). Not only is trehalose typically considered safe for cells, its protective properties under stressed conditions have been well documented (4). During dehydration process, trehalose can replace intracellular water molecules, maintain three-dimensional conformation of macromolecules, and transition into amorphous glass state to enable stable storage of live cells. Dried cells can potentially be stored above freezing temperatures, therefore eliminating the reliance on liquid nitrogen using current cryobanking methods (5). It has been demonstrated that the DNA integrity as well as nuclear structure and function of germinal vesicles (GVs), the nuclei of immature oocytes, can be maintained after dehydration in the presence of trehalose (6, 7). Dried-rehydrated GVs then need to be transferred to fresh ooplasm to reconstitute oocytes. Developing dry-preservation method for whole gametes, particularly the cumulus-oocyte complexes (COCs), will facilitate downstream *in vitro* fertilization (IVF) applications and bypass technical barriers and risks of GV transfer. However, while nuclei possess higher resilience to dehydration stress, dry-preservation of whole gametes remains challenging due to their high water and lipid contents and delicate cellular structures, such as membranous organelles and cytoskeleton.

Trehalose does not freely penetrate the cell membranes. To dry-preserve GVs, membranes permeabilization with chemicals, such as hemolysin, is required to incorporate trehalose into cells (8). When aiming at preserving whole gametes, safe and effective intracellular delivery of trehalose without permanent disruption of cell membrane is a critical first step. Trehalose hexaacetate (6-O-Ac-Tre) was engineered to increase the membrane permeability of the molecule. By replacing hydroxyl groups of trehalose with acetyl groups, the lipophilicity of the molecule increases and so does its membrane permeability (9). After entering the cells, 6-O-Ac-Tre can be deacetylated by non-specific esterases to convert it back to the non-modified form of trehalose with protective properties. Introducing trehalose intracellularly with 6-O-Ac-Tre therefore does not require any permanent or temporary disruption of cell membrane. Moreover, previous studies have demonstrated successful trehalose incorporation via 6-O-Ac-Tre in both somatic cells and oocytes before cryopreservation (10), making it an attractive option for developing dry-preservation methods for cumulus-oocyte complexes (COCs). While trehalose is generally considered safe for oocytes, the potential cytotoxicity of the engineered trehalose and its metabolic by-products after deacetylation remains to be assessed.

Microwave-assisted drying is one of the recent strategies to dry-preserve biomaterials. This method utilizes a low-level of microwave radiation to facilitate rapid and homogenous drying while maintaining samples within physiological temperatures (11). It has been applied to dry-preservation research on somatic cells, gametes, and gonadal tissues and demonstrated different levels of success (6, 12–15).

In the present study, we aimed to evaluate the feasibility of utilizing the membrane-permeable trehalose to improve dehydration tolerance of COCs using the domestic cat model. The conserved genetic and physiological traits in cats make them a valuable model species not only for wild feline but also other mammals, including humans (16). The objectives of the study were to (1) determine the efficacy and safety of trehalose delivery via 6-O-Ac-Tre, and (2) evaluate the effect of 6-O-Ac-Tre introduction on mitigating dehydration-induced cellular damages after microwave-assisted drying of COCs.

## 2 Materials and methods

All chemicals were purchased from Sigma-Aldrich unless otherwise indicated.

### 2.1 COC collection and culture

The Animal Care and Use Committee from the Smithsonian’s National Zoo and Conservation Biology Institute granted a waiver of the Animal Care and Use Committee for that study because cat ovaries and testes were collected at local veterinary clinics as byproducts from owner-requested routine spay and neuter. Ovaries from adult domestic cats were recovered after routine ovariohysterectomy and stored in Dulbecco’s PBS (DPBS) supplemented with 100 IU/mL penicillin and 100 μg/mL streptomycin at 4 °C until processing within 24 h. COCs were mechanically isolated into HMEM medium (HEPES-buffered MEM supplemented with 2 mM L-glutamine, 1 mM pyruvate, 100 IU/mL penicillin, 100 μg/mL streptomycin and 4 mg/mL bovine serum albumin [BSA]). COCs were selected based on their morphology. Only grade 1 (uniformly dark ooplasm, 5 or more layers of cumulus cells tightly packed around the oocyte) and grade 2 (same as grade 1, but with < 5 cell layers) COCs were collected based on standard classification criteria (17). To allow trehalose incorporation, COCs were incubated in 6-O-Ac-Tre at desired concentrations in MEM culture medium (MEM supplemented with 1 mM pyruvate, 2 mM L-glutamine, 100 IU/mL penicillin, 100 μg/mL streptomycin and 4 mg/mL BSA) for 4 or 24 h at 38.5 °C under a controlled humidified atmosphere with 5% CO_2_. Additionally, 50 μM of milrinone was added to the medium to prevent meiosis resumption during incubations (18).

### 2.2 Assessment of intracellular trehalose content

To measure trehalose content within COCs, 20 COCs per treatment per replicate were collected after incubating with desired concentrations of trehalose or 6-O-Ac-Tre (AAT Bioquest). COCs were washed with excess amount of DPBS 3 times to wash off any external trehalose or its derivative. COCs were then transferred to microcentrifuge tubes, snap frozen, and stored at −80 °C until ready for analysis. To extract trehalose, 50 μL of hot (80 °C) distilled water was added to each tube. COCs were lyzed by repeated frozen thaw three times before incubating at 80 °C for 10 min with occasional vortexing. Trehalose content was quantified with a trehalose assay kit (Megazyme) following manufacturer’s instruction. The kit utilized a series of enzymatic reactions to convert trehalose into reduced nicotinamide-adenine dinucleotide phosphate, which could be measured by the absorbance at 340 nm with a BioTek ELx808 microplate reader (BioTek Instruments). For assay controls, 0.5 mM of trehalose and 6-O-Ac-Tre were included to confirm that only the former was recognized by the enzyme. Given the number of cumulus cells may vary among COCs, relative trehalose content was calculated by normalizing trehalose content over protein concentration in each sample. Protein concentration was measured using Qubit protein assay kit following manufacturer’s instruction.

### 2.3 Assessment of oocyte survival

After culture, COCs were transferred to HMEM medium and mechanically denuded using a stripper micropipette. A cell-permeant dye Calcein AM (Invitrogen) that was converted into fluorescent Calcein by intracellular esterases was used to label live oocytes. A non-permeating dye propidium iodide (PI) was used to label dead or damaged cells. Oocytes were incubated with 2 μL/mL Calcein AM and 2 μL/mL of PI at 38.5 °C for 20 min. Fluorescent signals were visualized immediately with an Olympus BX41 epifluorescence microscope.

### 2.4 *In vitro* maturation (IVM), IVF, and embryo culture

**Figure 1:**
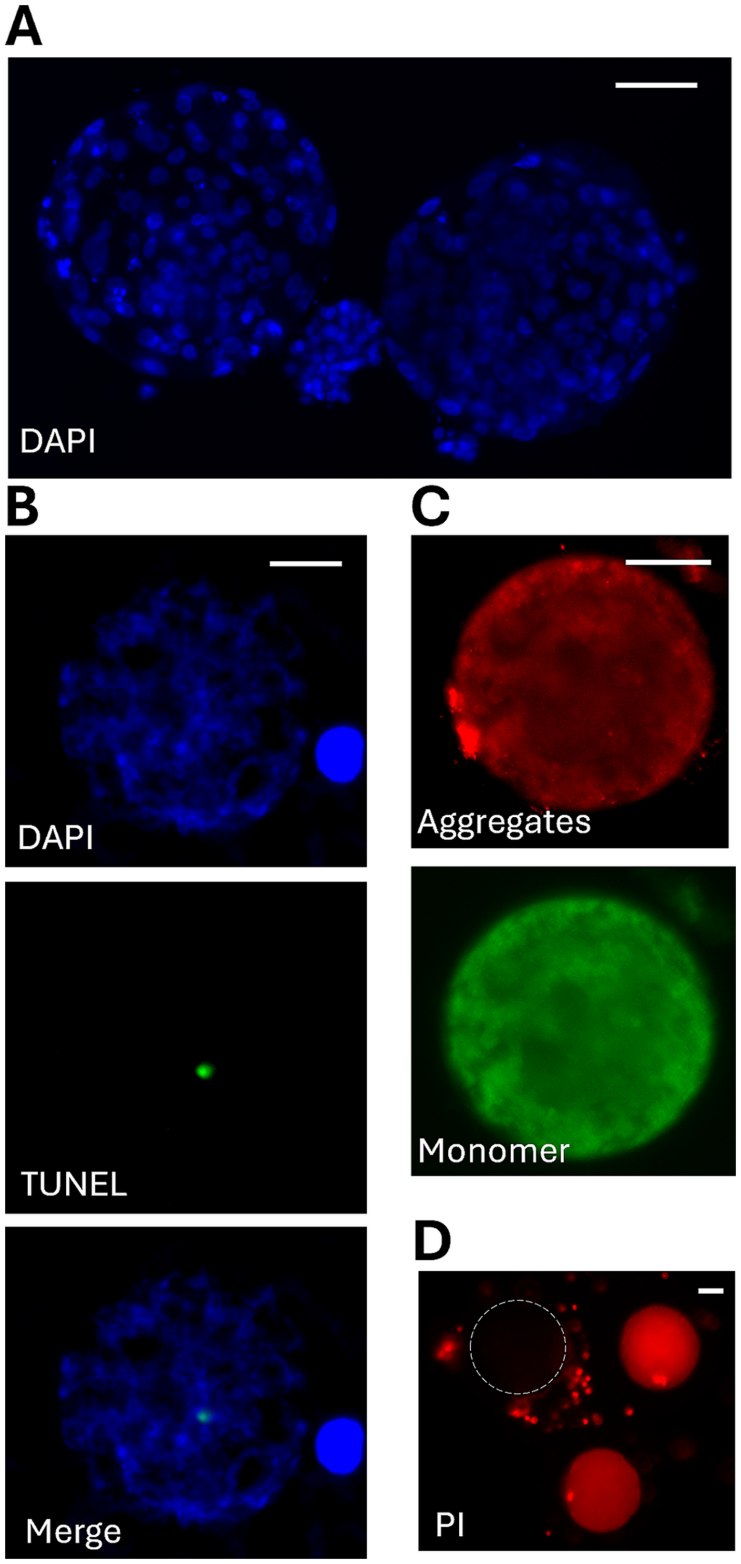
Representative images of different assessments. **(A)** Two blastocysts stained with DAPI. Scale bar: 50 μm. **(B)** Oocyte DNA integrity determined using TUNEL assay. DNA labelled with DAPI. TUNEL staining marked open ends of fragmented DNA. Scale bar: 20 μm. **(C)** Oocyte mitochondrial membrane potential (MMP) determined by JC-1 dye, which forms aggregates (red) when MMP is high and remains as monomers (green) when MMP is low. Scale bar: 50 μm. **(D)** Oocyte membrane integrity determined by non-permeable dye propidium iodide (PI) that marks dead or damaged cells (red). Circled outline labeled an oocyte with intact membrane. Scale bar: 50 μm.

For IVM, COCs were cultured in protein plus blastocyst medium (SAGE) containing 2 μg/mL of ovine LH (National Hormone and Pituitary Program) and 25 AU/L of porcine FSH in 50 μL microdrops. The COCs were cultured in 38.5 °C incubators with 5% CO_2_ for 26 h. For IVF, COCs were incubated with 1 × 10^6^/mL of cat epididymal spermatozoa in protein plus blastocyst medium for 20 h. COCs then were mechanically denuded before culturing in in blastocyst medium in 38.5 °C incubators with 5% CO_2_. Non-cleaved oocytes were removed from embryo culture and fixed in 4% paraformaldehyde (PFA). Remaining embryos were cultured for up to 7 days before fixation. The fixed samples were then mounted with Vectashield mounting medium with DAPI (Vector Labs). Maturation of oocytes was determined by assessing chromosomal configuration and alignment as well as the presence of polar bodies. An embryo with a visible blastocoele and at least 64 blastomeres was considered a blastocyst (Figure 1A).

### 2.5 Microwave-assisted drying and rehydration of COCs

Microwave drying was adapted from previously described procedures (8). Trehalose-loaded COCs were transferred to 0.3 M trehalose in Tris-EDTA buffer. Suspension of COCs (up to 10) in 20 μL trehalose solution was deposited on a piece of ¾ × ¾ inch of weighing paper (Fisher Scientific) and dried for 0, 10 or 15 min in a SAM 255 microwave (CEM) at 20% power with upper temperature threshold set at 40 °C. To rehydrate, 50 μL of 0.3 M trehalose solution was deposited on top of the samples and incubated at 38.5 °C for 5 min, followed by adding 50 μL of HMEM directly to the solution to reach a trehalose concentration of 0.15 M and incubating for another 5 min. COCs were then transferred to HMEM and allowed to recover for 20 min before assessment.

### 2.6 Assessment of DNA integrity

DNA fragmentation was detected by terminal deoxynucleotidyl transferase dUTP nick end labeling (TUNEL) assay using an *in situ* cell death detection kit (Roche Applied Science). COCs were denuded and fixed in 4% PFA at 4 °C overnight. After rinsing off PFA with DPBS, oocytes were permeabilized with 0.5% Triton X-100 solution for 30 min at room temperature before exposure to TUNEL reaction mixture for 1 h at 38 °C according to the manufacturer’s instruction. Oocytes were then washed three times in DPBS and mounted on slides with Vectashield mounting medium containing DAPI. Fluorescent signals were visualized with the Olympus BX41 epifluorescence microscope (Figure 1B). DNA with any positive TUNEL signal was considered damaged.

### 2.7 Assessment of mitochondrial membrane potential and cell membrane integrity

JC-1 dye (Invitrogen) was used to assess mitochondrial membrane potential. In healthy mitochondria with high membrane potential, JC-1 accumulates in mitochondria forming red fluorescent aggregates (J-aggregates). Conversely, mitochondrial depolarization results in more JC-1 remaining as green fluorescent monomer. The ratio of red to green fluorescence serves as an indicator of mitochondrial health. COCs were denuded and incubated with 1 μL/mL of JC-1 probe at 38.5 °C for 30 min. Fluorescent signals were visualized immediately with an Olympus BX41 epifluorescence microscope. Images of both red and green fluorescence were taken under same exposure time using Zen software (v3.6; Zeiss Microscopy) (Figure 1C). Fluorescent intensities of red and green signals were measured using ImageJ software (National Institutes of Health). To assess cell membrane integrity, the non-permeating dye PI was used to label cells with damaged membrane (Figure 1D), as described earlier.

### 2.8 Experimental design

First, we aimed to validate the incorporation of trehalose into COCs via 6-O-Ac-Tre. COCs (*N* = 1,000 in 5 replicates) collected from adult cats were co-incubated with 0, 3, 10, or 30 mM of 6-O-Ac-Tre at 38.5 °C for 4 h or overnight. Samples were collected and intracellular trehalose content was measured with trehalose assay kit. Next, we examined potential cytotoxicity from the exposure to 6-O-Ac-Tre. COCs were co-incubated with 0, 3, 10, or 30 mM of 6-OAc-Tre at 38.5 °C overnight. Oocyte survival (*N* = 185 COCs from 37 cats in 3 replicates) and their meiotic and developmental competence (*N* = 519 COCs from 102 cats in 7 replicates) were assessed. Based on the findings from the cytotoxic assessment, we then investigated the effect the two non-cytotoxic doses (3 and 10 mM) of 6-O-Ac-Tre on dehydration tolerance of COCs. COCs were co-incubated with 0, 3, or 10 mM of 6-O-Ac-Tre at 38.5 °C overnight. They were then microwave-dried in the presence of 0.3 M external trehalose for either 10 or 15 min (roughly corresponding to removal of 75 and 96% of water). COCs cultured without 6-O-Ac-Tre (0 mM) and did not undergo microwave-drying (0 min) were used as controls. Immediately after drying, COCs were rehydrated. Integrity or health of cellular components prone to dehydration injuries were then assessed, including DNA (N = 169 COCs from 64 cats in 5 replicates), mitochondria (*N* = 245 from 59 cats in 5 replicates), and cell membrane (*N* = 228 from 43 cats in 4 replicates). Functional assessment of the maturation ability of dried/rehydrated COCs was also performed (*N* = 303 COCs from 32 cats in 3 replicates).

### 2.9 Statistical analysis

For quantitative results (JC-1 intensity), each data point represented measurements obtained from individual oocyte. For qualitative results (oocyte survival, maturation, cleavage, blastocyst formation, DNA and membrane integrity), each data point represented percentage within each replicate. Data were assessed by Shapiro–Wilk tests to determine normality. Data following normal distribution were analyzed by analysis of variance (ANOVA) followed by Tukey’s multiple test. Others were analyzed by Kruskal-Wallis test followed by Dunn’s test. Differences were considered significant at *p* < 0.05 (GraphPad Prism 7.03; GraphPad Software).

## 3 Results

### 3.1 Incorporation of intracellular trehalose in COCs via exposure with 6-O-Ac-Tre

**Figure 2:**
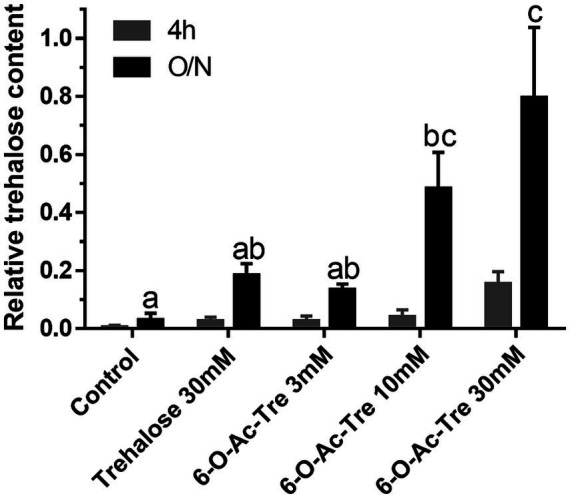
Trehalose incorporation into cat COCs. Relative trehalose content (mean ± SEM) after 4 h or overnight (O/N) incubation with trehalose or 6-O-Ac-Tre. No significant difference was observed among 4 h incubation groups. Among O/N groups, values with different letters differ, *p* < 0.05.

To determine if trehalose hexaacetate was incorporated into COCs and converted to trehalose, assays were conducted to measure intracellular trehalose content. Intracellular trehalose was below the detection limit of the assay in any group after 4 h of incubation with 6-O-Ac-Tre (Figure 2). With overnight incubation, intracellular trehalose increased in the COCs incubated with 6-O-Ac-Tre in a dose-dependent manner. COCs incubated with either 10 mM or 30 mM of 6-O-Ac-Tre contained significantly higher (*p* < 0.05) amount of trehalose compared to those incubated with 3 mM 6-O-Ac-Tre or 30 mM of the non-modified trehalose (Figure 2). These data suggest that 6-O-Ac-Tre can indeed effectively enter the COCs and be converted into regular trehalose.

### 3.2 Cytotoxicity assessment of 6-O-Ac-Tre on COCs

**Figure 3:**
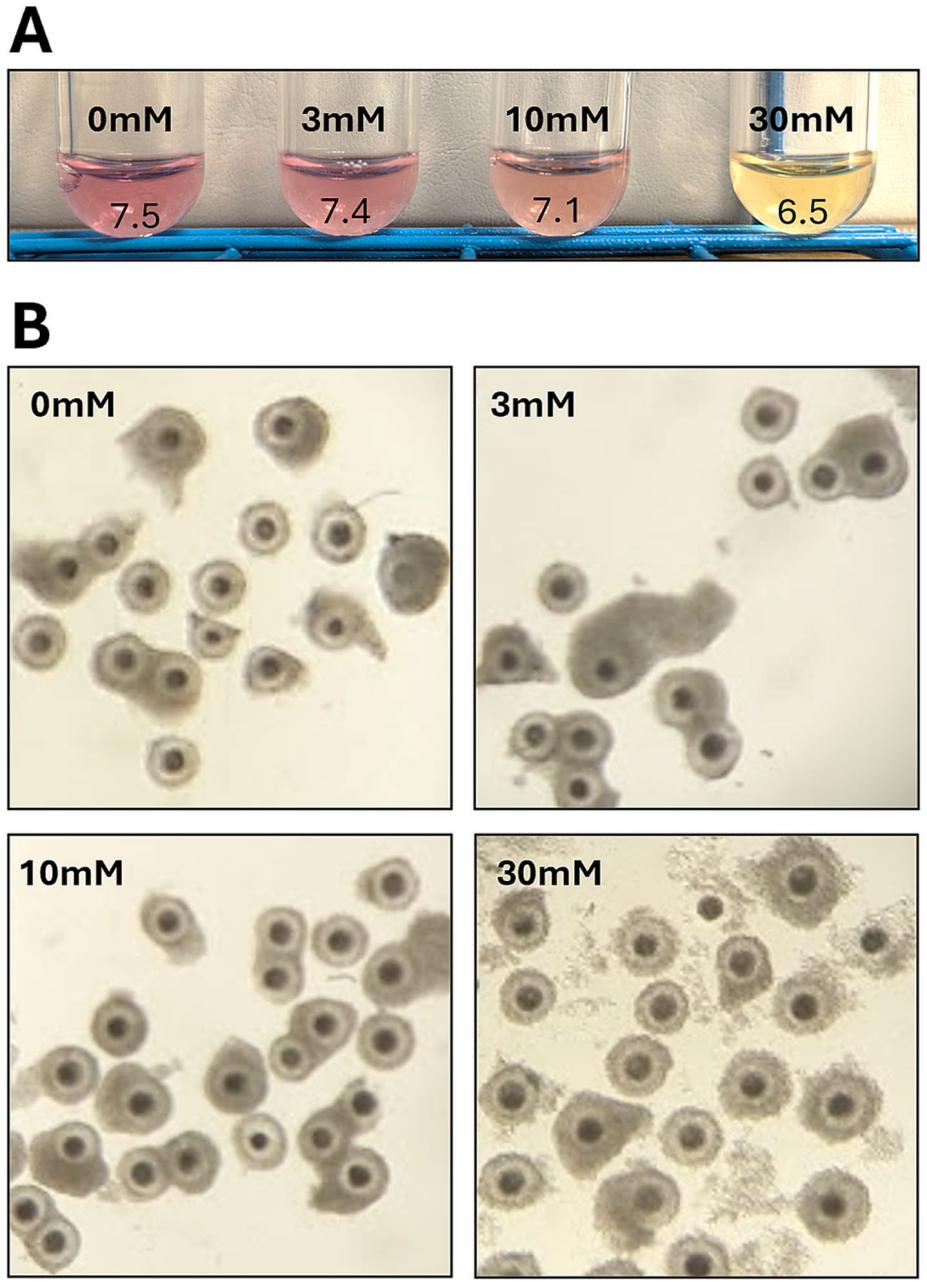
Culture media and COC morphology after overnight incubation with different concentrations of 6-O-Ac-Tre. **(A)** Difference in the pH of culture media based on phenol red indicator phenol red indicator and pH strips. **(B)** Representative images of COC morphology after overnight culture.

In culture medium without 6-O-Ac-Tre (controls), pH values remained between 7.4 and 7.5 after overnight incubation. However, a decrease in the pH value of medium containing 6-O-Ac-Tre was observed after overnight incubation, as indicated by phenol red in the culture medium (Figure 3A). Using pH strips, media supplemented with 3 mM 6-O-Ac-Tre showed similar pH values around 7.4 post-incubation. However, pH values in media containing 10 or 30 mM 6-O-Ac-Tre were around 7.1 and 6.5, respectively. Similar acidification was also observed in media without COCs. COC morphology appeared normal with tightly packed cumulus cells surrounding oocytes after overnight incubation in 0, 3, and 10 mM 6-O-Ac-Tre. After overnight incubation with 30 mM 6-O-Ac-Tre, loose or dissociated cumulus cells were observed after overnight incubation (Figure 3B). These observations indicated a potential cytotoxicity of 6-O-Ac-Tre exposure, and resulting media acidification, over longer incubation times.

**Figure 4:**
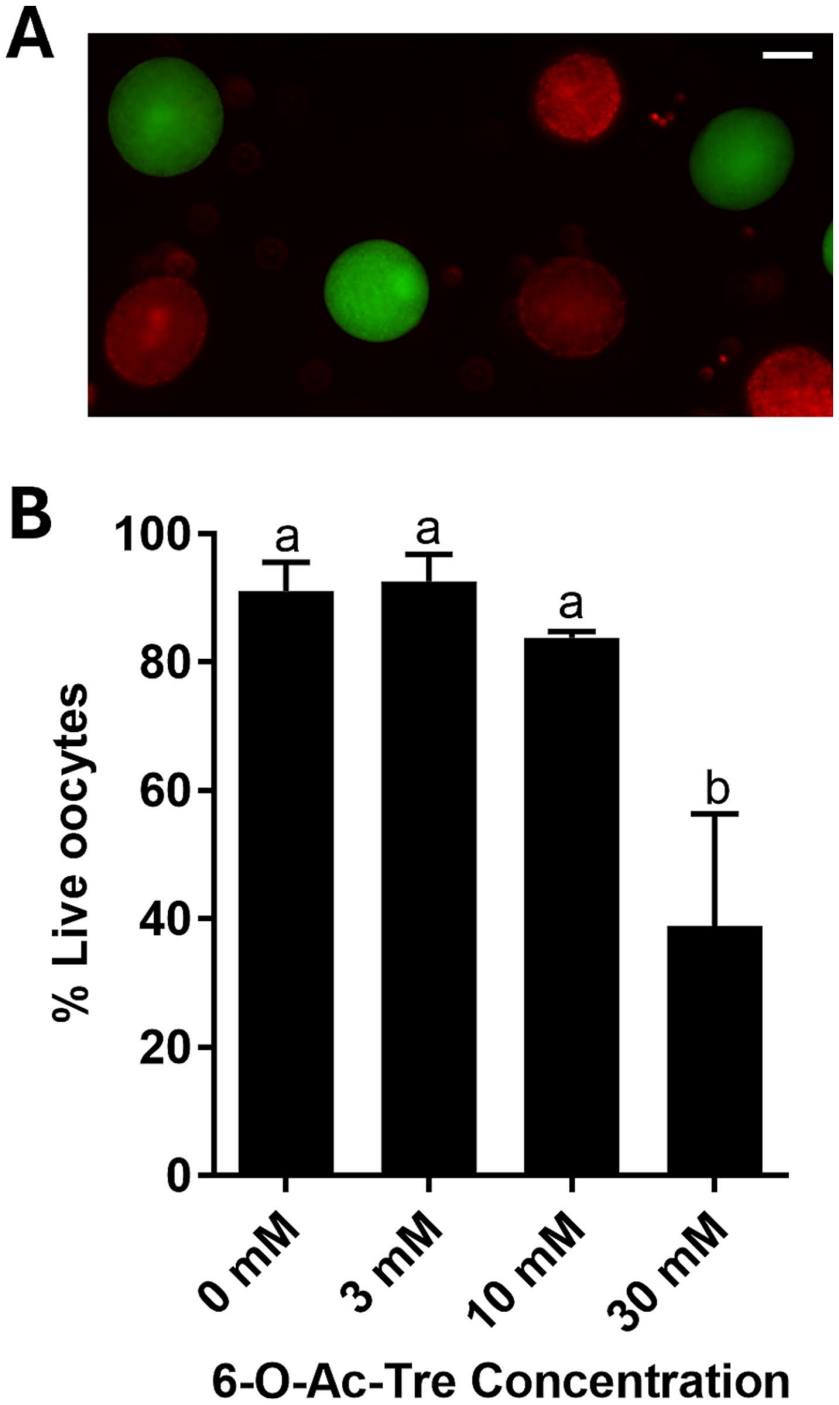
Assessment of oocyte survival after overnight incubation of COCs with different concentrations of 6-O-Ac-Tre. **(A)** Representative image of live/dead assay. Calcein marks live cells (green) and propidium iodide labels dead or damaged cells (red). Scale bar: 50 μm. **(B)** Percentage of live oocytes evaluated by live/dead assay. Values (mean ± SEM) with different letters differ, *p* < 0.05.

**Figure 5:**
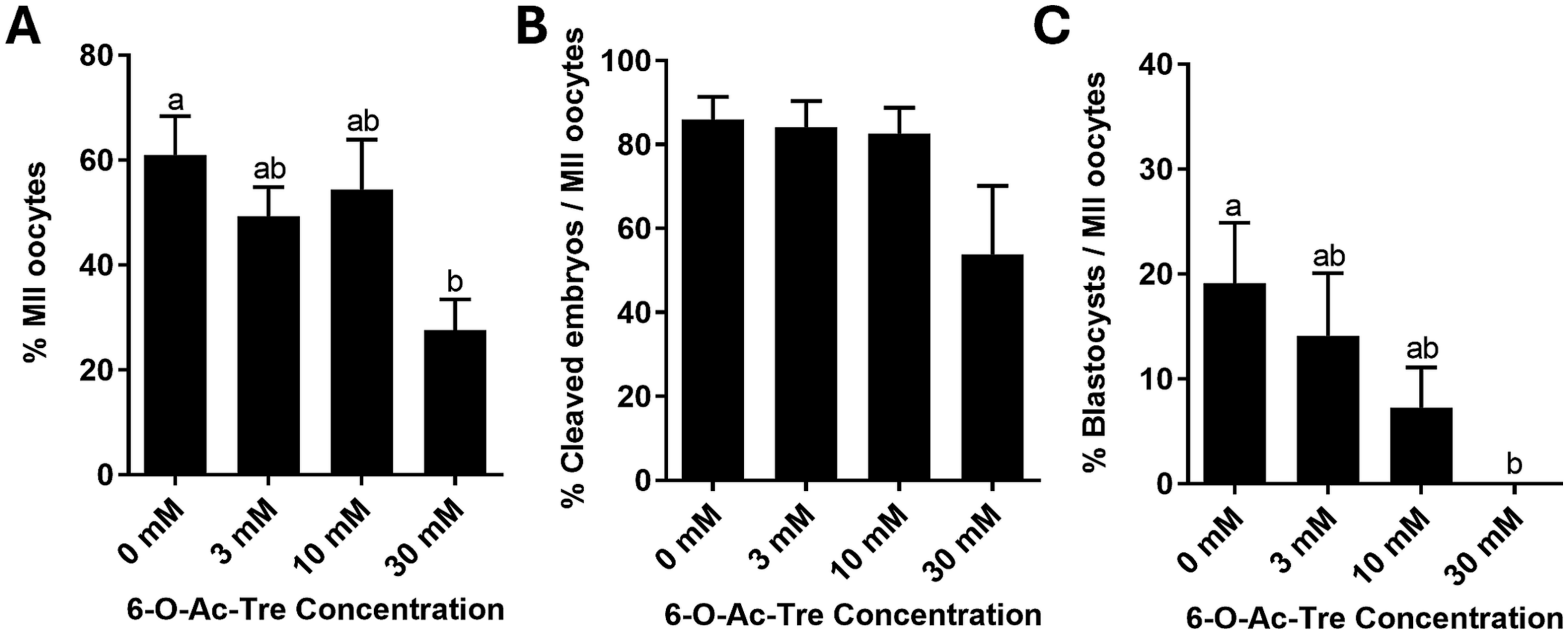
Assessment of meiotic and developmental competence of COCs after overnight incubation with different concentrations of 6-O-Ac-Tre. **(A)** Percentage of oocytes reaching metaphase II (MII) after *in vitro* maturation. **(B,C)** Percentages of mature oocytes that cleaved or formed blastocyst after *in vitro* fertilization. Values (mean ± SEM) with different letters differ, *p* < 0.05.

Impact of overnight incubation with 6-O-Ac-Tre on oocyte survival was further investigated using live/dead assays (Figure 4A). Overnight incubation with either 3 or 10 mM of 6-O-Ac-Tre did not affect oocyte survival (92.7 ± 4.1% and 83.8 ± 1.0%, respectively, *p* > 0.05), compared to the control group without any engineered trehalose (91.0 ± 4.5%). However, overnight exposure to 30 mM of 6-O-Ac-Tre led to a significant reduction of the percentage of live oocytes (38.9 ± 17.5%, *p* < 0.05; Figure 4B). IVM and IVF were then performed to assess the functionality of COCs after 6-O-Ac-Tre incubation. COCs exposed to 3 or 10 mM 6-O-Ac-Tre maintained their ability to mature (49.3 ± 5.5% and 54.3 ± 9.6%, respectively), cleave (84.1 ± 6.3% and 82.6 ± 6.1% of mature oocytes, respectively) and form blastocysts (14.1 ± 6% and 7.3 ± 3.8% of mature oocytes), compared to the control group (60.9 ± 7.5% mature, 86.0 ± 5.3% cleaved, and 19.1 ± 5.8% formed blastocysts, Figure 5). Maturation capacity of COCs incubated with 30 mM 6-O-Ac-Tre was compromised (*p* < 0.05) with only 27.6 ± 5.9% of oocytes reaching metaphase II (MII). Of the matured COCs, 53.8 ± 16.4% cleaved, indicating a moderate yet not significantly different decline (*p* > 0.05). Furthermore, none of the embryos reached blastocyst stage in this group (Figure 5). Thus, we concluded that 30 mM 6-O-Ac-Tre was cytotoxic to COCs whereas the two lower concentrations appeared safer to use on these cells.

### 3.3 Assessment of dehydration tolerance of COCs after exposure to 6-O-Ac-Tre

**Figure 6:**
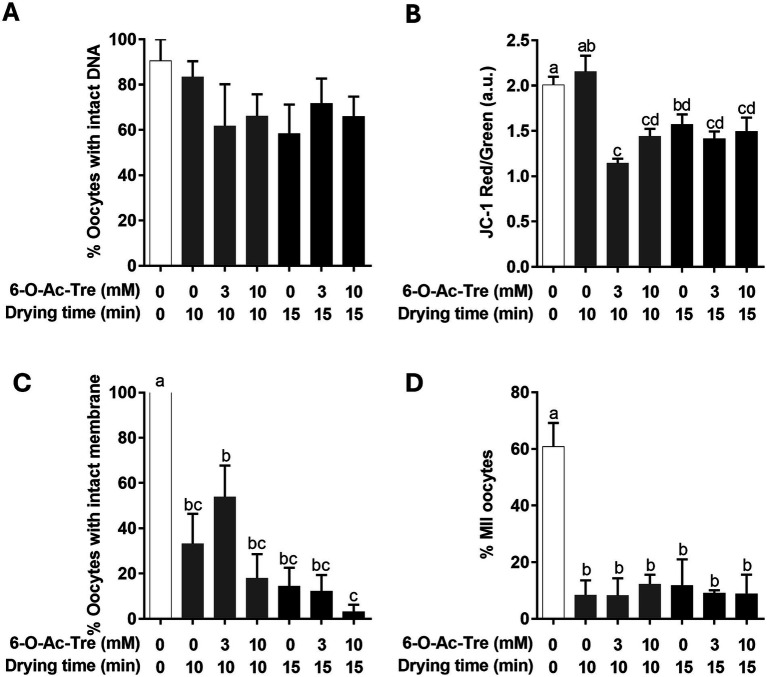
Assessment of cellular components within oocytes and meiotic competence of COCs after microwave drying and rehydration. **(A)** Percentage of oocytes with intact DNA evaluated by TUNEL assay. **(B)** Ratio of red/green JC-1 signal as an indicator for mitochondrial membrane potential. **(C)** Percentages of oocytes with intact membrane evaluated with PI dye. **(D)** Percentages of MII oocytes after in vitro maturation. Values (mean ± SEM) with different letters differ, *p* < 0.05.

By examining COC cellular structures known to be vulnerable to dehydration stress, the protective of trehalose incorporation via 6-O-Ac-Tre was characterized. While the percentages of oocytes with intact DNA appeared modestly lower in all treatment groups after drying and rehydration (range, 58.3 to 83.3%) compared to the control group that was not exposed to 6-O-Ac-Tre or underwent microwave drying (90.5 ± 9.5%; Figure 6A), none of the differences reached statistical significance (*p* > 0.05). In the group of oocytes without 6-O-Ac-Tre incorporation (0 mM), mitochondrial membrane potential within the oocytes was not affected after 10 min of drying. However, significant reduction (*p* < 0.05) was observed after 15 min of drying. In the oocytes incubated with 3 or 10 mM 6-O-Ac-Tre, decreased mitochondrial membrane potential (*p* < 0.05) was observed after both 10 and 15 min of drying, compared to the control group (Figure 6B). Examination of oocytes’ membrane integrity showed that percentages of oocytes with intact membrane dramatically decreased (p < 0.05) in all the treatment groups, compared to 100% membrane integrity in the controls. Without trehalose incorporation, only 33.2 ± 13.2% of the oocytes maintained intact membrane after 10 min of drying. Percentage of oocytes with intact membrane dropped to 14.3 ± 8.3% after 15 min of drying. Incorporation of engineered trehalose, regardless of concentration, was not able to mitigate the damage. Of oocytes incubated with 3 mM 6-O-Ac-Tre, 53.9 ± 13.8% and 12.2 ± 7.2% maintained intact membrane after 10 and 15 min of drying, respectively. Percentages of oocytes with intact membrane after 10 and 15 min of drying were 17.9 ± 10.7% and 3.1 ± 3.1%, respectively, among those incubated with 10 mM 6-O-Ac-Tre (Figure 6C). Importantly, dehydration impaired meiotic maturation of oocytes. Percentages of oocytes reaching MII decreased drastically from 60.8 ± 8.3% to below 12.2% (range, 8.2 to 12.2%) after 10 or 15 min of drying, regardless of the presence of absence of trehalose (Figure 6D).

## 4 Discussion

Trehalose is a well-recognized protectant for cryopreservation and dry-preservation, attributing to its superior property to vitrify after freezing or dehydration. While it has been established that cell membrane is impermeable to trehalose, it has been shown that low levels of trehalose can enter cells through endocytosis over long exposure time (19). We also detected low levels of intracellular trehalose in COCs after overnight, but not 4 h of incubation, likely through the same mechanism. 6-O-Ac-Tre was engineered to increase the permeability of the sugar through the cell membrane. Initial study reported that intracellular trehalose accumulation in rat hepatocytes was detectable after incubating in 30 mM of 6-O-Ac-Tre for 3 h (9). In the present study, no significant increase of intracellular trehalose was detected in cat COCs after 4 h of incubation with 30 mM 6-O-Ac-Tre. Elevation of intracellular trehalose was detected in a dose-dependent manner after overnight incubation, consistent with a previous report (10). COCs incubated with 30 mM of 6-O-Ac-Tre contained more than 4-fold of intracellular trehalose than those incubated with same concentration of non-modified trehalose. Even at the lower concentration (3 mM), 6-O-Ac-Tre incubation resulted in similar level of intracellular trehalose as what was detected after overnight incubation with 30 mM trehalose. A previous study also confirmed the presence of trehalose within ooplasm after incubating cat COCs with 3 to 30 mM of 6-O-Ac-Tre for at least 12 h (10). Taken together, our findings confirm that the engineered trehalose indeed facilitates the incorporation and accumulation of intracellular trehalose in COCs.

The acidification of culture media containing 6-O-Ac-Tre after incubation was unexpected, as it has not been specifically reported in previous studies using this modified molecule (9, 10). Hydrolysis of 6-O-Ac-Tre by esterases results in the production of trehalose and acetic acid, which will decrease the pH value. However, the acidification we observed was also seen in media free of cells, suggesting that it was independent of endogenous esterase activity. There are two potential sources for the speculated hydrolysis reaction. First, 6-O-Ac-Tre may undergo non-enzymatic deacetylation. While the rate of such spontaneous reaction is typically considered negligible in neutral conditions, it may accumulate over long incubation time. Alternatively, minute esterase activity may exist in the culture medium supplemented with various components from different sources. In either scenario, the initial minuscule amount of acetic acid produced from the reaction may create a slightly more acidic environment, which in turn may accelerate the hydrolysis reaction and further amplify the acidification effect after overnight incubation (20, 21). This hypothesis remains speculative and more investigation is needed to identify the actual source of the acidification.

Cytotoxicity assessments suggested that both cat COCs exposure to 3 and 10 mM of 6-O-Ac-Tre were safe, whereas 30 mM 6-O-Ac-Tre reduced both the survival and competence of the oocytes. This is consistent with previous report that feline COCs incubated with 15 or 30 mM 6-O-Ac-Tre for 24 h showed significantly lower maturation and fertilization rates (10). The adverse effect could at least partially be attributed to the acidification of culture media. COCs are known to possess regulatory mechanisms to maintain intracellular pH, allowing them to tolerate mild change in environmental pH (22). However, suboptimal pH could compromise oocyte quality by disrupting meiotic spindle assembly and calcium oscillation patterns of the oocytes, which in turn lead to decreased meiotic and developmental competence (23–25). More severe acidic stress could even induce cell death (26). The cytotoxicity of higher concentrations of 6-O-Ac-Tre could be a limiting factor for the application of this engineered molecule, especially when high intracellular trehalose is desired.

Trehalose has been shown to mitigate dehydration-induced cellular damages in gametes (3). Previous studies demonstrated that DNA integrity was largely maintained in germinal vesicles of cat oocytes for at least 8 weeks after drying and storage in the presence of both extra- and intracellular trehalose (7, 27, 28). In the present study, the decrease in the percentage of oocytes with intact DNA after microwave drying was modest and not statistically significant. While the results were consistent with the previous research, it should be noted that the lack of statistical significance may reflect high individual difference and limited statistical power. Interestingly, even the COCs without 6-O-Ac-Tre incubation were able to maintain DNA integrity after dehydration and immediate rehydration. This result suggests that the presence of external trehalose, without intracellular incorporation, was sufficient to protect DNA integrity in the GV oocytes under current drying conditions. This could reflect the higher tolerance of GV oocytes to non-physiological conditions (29). Previous studies showed that DNA damage increased with microwave time and storage time (7, 28). The effect of intracellular trehalose on the stability of DNA over longer drying and storage time remained to be examined.

In previous studies, drying led to reduced mitochondrial membrane potential and active mitochondria in sperm and somatic cells (30, 31). Here, we reported that without incorporation of intracellular trehalose, mitochondrial membrane potential in cat COCs was unaffected after 10 min of microwaving. This suggests that mitochondria in these cells were capable of tolerating partial dehydration for up to 75% water removal. However, COCs pre-incubated with 6-O-Ac-Tre possessed lower mitochondrial membrane potential after the same drying time. It has been shown that trehalose protected the integrity and function of mitochondria after freezing or dehydration (32, 33). Therefore, introducing intracellular trehalose is less likely the cause of the decreased mitochondrial membrane potential. We speculate that the effect may be a manifestation of the acidic culture condition created by 6-O-Ac-Tre. Regulation of intracellular pH of growing oocytes is facilitated by their surrounding cells (22), which likely helps to maintain normal function of COCs exposed to 3 or 10 mM 6-O-Ac-Tre. However, dehydration is known to damage cytoskeleton, causing disruption of actin-based communication between oocytes and cumulus cells (34, 35). It is plausible that the impaired cell–cell communications might have undermined the homeostasis within the oocytes, leaving them more vulnerable to dehydration stress. This explanation remains speculative and requires more future study to investigate the underlying mechanisms. By 15 min of microwave-drying, mitochondria in the oocytes, regardless of 6-O-Ac-Tre exposure, showed decreased membrane potential, confirming the detrimental effect of dehydration stress on mitochondrial health. Taken together, our data suggest that rather than mitigating the damage, pre-incubation with 6-O-Ac-Tre further exacerbated the dehydration tolerance of the mitochondria.

Plasma membrane is the cell’s first line of defense against environmental disturbance and one of the most sensitive cellular components to dehydration stress (3). It is one of the main obstacles in developing successful dry-preservation techniques beyond the nuclei. Indeed, drastic decrease in membrane integrity was readily observed in oocytes without intracellular trehalose after partial dehydration. It has been proposed that trehalose protects membranes by replacing water molecules in the lipid bilayers and vitrifying into amorphous glass during dehydration, preventing collapsing and maintaining fluidity of the lipid structure (36, 37). If the membrane injuries can be minimized, cells possess multiple mechanisms to repair the damages (38). However, our results showed that introducing intracellular trehalose via 6-O-Ac-Tre into COCs did not sufficiently mitigate the detrimental effect on membrane integrity. Dehydration stress likely induces damage on both the membrane and the proteins involved in membrane repair (3, 38). Extensive membrane breach could trigger a cascade of cytosolic events that lead to cell death. Additional trehalose or other protective agents will be required to provide better shield against dehydration-induced membrane injuries to ensure cell survival.

Consistent with previous studies, our results showed that dehydration of COCs without protection from internally-incorporated trehalose was detrimental to their ability to mature (39). No improvement was found with introduction of trehalose via 6-O-Ac-Tre. Oocyte maturation is a concerted event involving a multitude of molecules and cellular components. Damage to any of these factors could reduce the meiotic capability of the oocytes. A combination of structural and molecular injuries induced by dehydration stress, including the ones demonstrated in this study, likely contributed to the drastic decrease in maturation rate after only 10 min of microwave drying. On a more positive note, a small number of COCs not only survived 15-min of drying but were also capable to mature afterwards. While it is unclear what combination of intrinsic and extrinsic factors led to the endurance of these individual COCs, it nonetheless demonstrated encouraging potential for dry-preservation.

Taken together, our findings raise concerns on the safety and effectiveness of utilizing 6-O-Ac-Tre to introduce intracellular trehalose into COCs to enhance their dehydration tolerance. Incorporation of intracellular trehalose remains the priority task for developing dry-preservation methods. Other trehalose delivery approaches, such as temporary phospholipid-phase transition or biocompatible vectors, should be explored (40). Ultimately, employing a safe method to effectively introduce trehalose into cells can widely benefit long-term biopreservation efforts at ambient temperatures that are more affordable and sustainable.

## Data Availability

The raw data supporting the conclusions of this article will be made available by the authors, without undue reservation.
